# The emerging role of Nrf2 in mitochondrial function

**DOI:** 10.1016/j.freeradbiomed.2015.04.036

**Published:** 2015-11

**Authors:** Albena T. Dinkova-Kostova, Andrey Y. Abramov

**Affiliations:** a Jacqui Wood Cancer Centre, Division of Cancer Research, Medical Research Institute, University of Dundee, Dundee DD1 9SY, Scotland, UK; b Departments of Medicine and Pharmacology and Molecular Sciences, Johns Hopkins University School of Medicine, Baltimore, MD 21205, USA; c Department of Molecular Neuroscience, University College London Institute of Neurology, London WC1N 3BG, UK

**Keywords:** Bioenergetics, Cytoprotection, Keap1, Mitochondria, Nrf2, Free radicals

## Abstract

The transcription factor NF-E2 p45-related factor 2 (Nrf2; gene name *NFE2L2*) allows adaptation and survival under conditions of stress by regulating the gene expression of diverse networks of cytoprotective proteins, including antioxidant, anti-inflammatory, and detoxification enzymes as well as proteins that assist in the repair or removal of damaged macromolecules. Nrf2 has a crucial role in the maintenance of cellular redox homeostasis by regulating the biosynthesis, utilization, and regeneration of glutathione, thioredoxin, and NADPH and by controlling the production of reactive oxygen species by mitochondria and NADPH oxidase. Under homeostatic conditions, Nrf2 affects the mitochondrial membrane potential, fatty acid oxidation, availability of substrates (NADH and FADH_2_/succinate) for respiration, and ATP synthesis. Under conditions of stress or growth factor stimulation, activation of Nrf2 counteracts the increased reactive oxygen species production in mitochondria via transcriptional upregulation of uncoupling protein 3 and influences mitochondrial biogenesis by maintaining the levels of nuclear respiratory factor 1 and peroxisome proliferator-activated receptor γ coactivator 1α, as well as by promoting purine nucleotide biosynthesis. Pharmacological Nrf2 activators, such as the naturally occurring isothiocyanate sulforaphane, inhibit oxidant-mediated opening of the mitochondrial permeability transition pore and mitochondrial swelling. Curiously, a synthetic 1,4-diphenyl-1,2,3-triazole compound, originally designed as an Nrf2 activator, was found to promote mitophagy, thereby contributing to the overall mitochondrial homeostasis. Thus, Nrf2 is a prominent player in supporting the structural and functional integrity of the mitochondria, and this role is particularly crucial under conditions of stress.

## Highlights

• Nrf2 has a crucial role in maintaining cellular redox homeostasis.
• Nrf2 affects the mitochondrial membrane potential and ATP synthesis.
• Nrf2 influences mitochondrial fatty acid oxidation.
• Nrf2 supports the structural and functional integrity of the mitochondria.
• Nrf2 activators have beneficial effects when mitochondrial function is compromised.

## Introduction

**Fig. 1:**
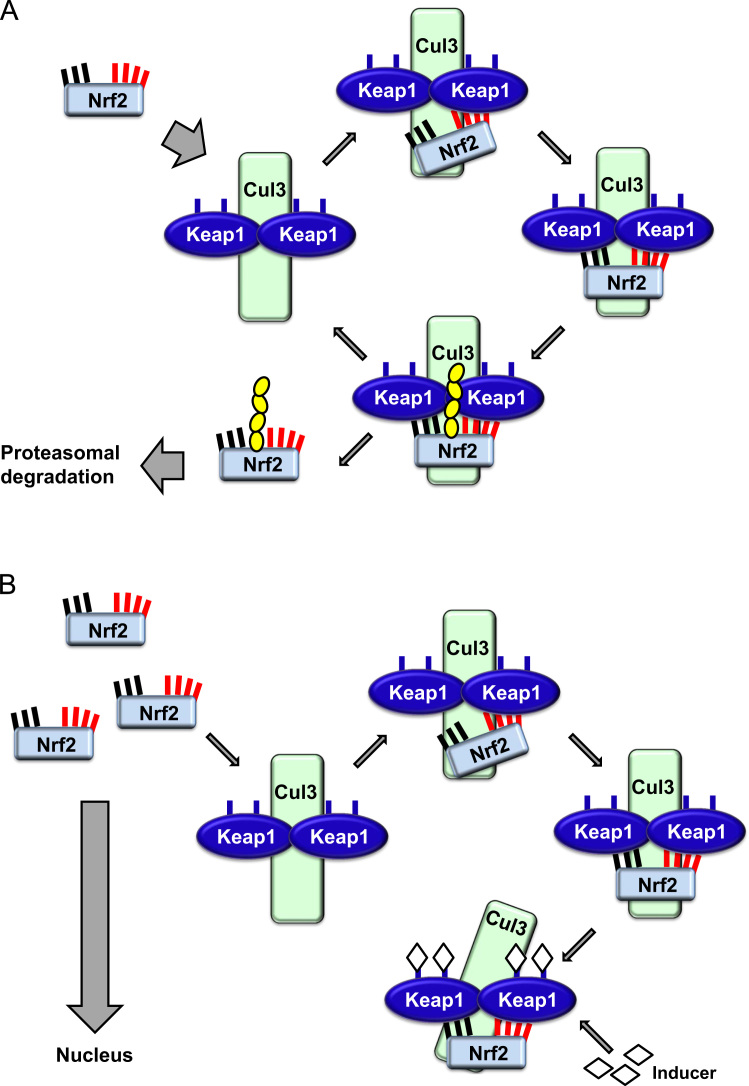
The cyclic sequential binding and regeneration model for Keap1-mediated degradation of Nrf2. (A) Nrf2 binds sequentially to a free Keap1 dimer: first through its high-affinity ETGE (red sticks) binding domain and then through its low-affinity DLG (black sticks) binding domain. In this conformation of the protein complex, Nrf2 undergoes ubiquitination and is targeted for proteasomal degradation. Free Keap1 is regenerated and able to bind to newly translated Nrf2, and the cycle begins again.(B) Inducers (white diamonds) react with sensor cysteines of Keap1 (blue sticks), leading to a conformational change and impaired substrate adaptor activity. Free Keap1 is not regenerated, and the newly synthesized Nrf2 accumulates and translocates to the nucleus.

The transcription factor NF-E2 p45-related factor 2 (Nrf2; gene name *NFE2L2*) regulates the expression of networks of genes encoding proteins with diverse cytoprotective activities. Nrf2 itself is controlled primarily at the level of protein stability. Under basal conditions, Nrf2 is a short-lived protein that is subjected to continuous ubiquitination and proteasomal degradation. There are three known ubiquitin ligase systems that contribute to the degradation of Nrf2. Historically, the first negative regulator of Nrf2 to be discovered was Kelch-like ECH-associated protein 1 (Keap1) [1], a substrate adaptor protein for Cullin 3 (Cul3)/Rbx1 ubiquitin ligase [2], [3], [4]. Keap1 uses a highly efficient cyclic mechanism to target Nrf2 for ubiquitination and proteasomal degradation, during which Keap1 is continuously regenerated, allowing the cycle to proceed (Fig. 1A) [5]. Nrf2 is also subjected to degradation mediated by glycogen synthase kinase (GSK)3/β-TrCP-dependent Cul1-based ubiquitin ligase [6], [7]. Most recently, it was reported that, during conditions of endoplasmic reticulum stress, Nrf2 is ubiquitinated and degraded in a process mediated by the E3 ubiquitin ligase Hrd1 [8].

In addition to serving as a ubiquitin ligase substrate adaptor protein, Keap1 is also the sensor for a wide array of small-molecule activators of Nrf2 (termed inducers) [9]. Inducers block the cycle of Keap1-mediated degradation of Nrf2 by chemically modifying specific cysteine residues within Keap1 [10], [11] or by directly disrupting the Keap1:Nrf2 binding interface [12], [13]. Consequently, Nrf2 is not degraded, and the transcription factor accumulates and translocates to the nucleus (Fig. 1B), where it forms a heterodimer with a small Maf protein; binds to antioxidant-response elements, the upstream regulatory regions of its target genes; and initiates transcription [14], [15], [16]. The battery of Nrf2 targets comprises proteins with diverse cytoprotective functions, including enzymes of xenobiotic metabolism, proteins with antioxidant and anti-inflammatory functions, and proteasomal subunits, as well as proteins that regulate cellular redox homeostasis and participate in intermediary metabolism.

## Nrf2, a master regulator of cellular redox homeostasis

**Fig. 2:**
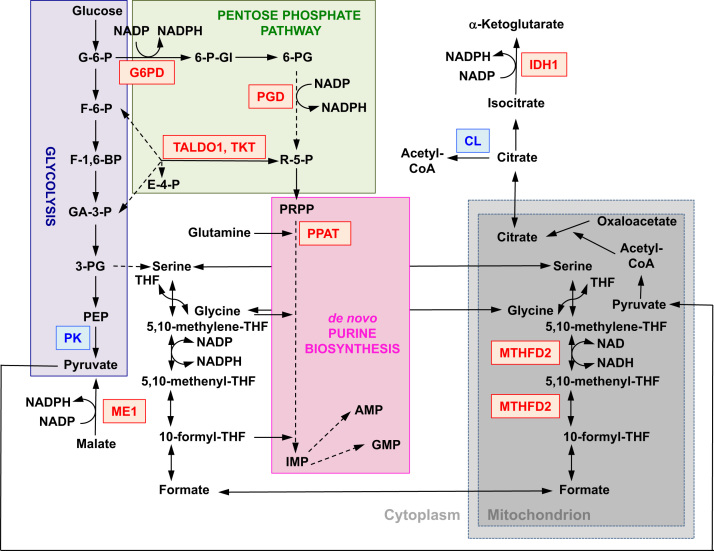
The role of Nrf2 in the metabolism of rapidly proliferating cells. Nrf2 is a positive regulator of genes encoding enzymes in both the oxidative arm [i.e., glucose-6-phosphate dehydrogenase (G6PD) and 6-phosphogluconate dehydrogenase (PGD)] and the nonoxidative arm [i.e., transaldolase 1 (TALDO1) and transketolase (TKT)] of the pentose phosphate pathway. G6PD and PGD generate NADPH. Nrf2 also regulates the gene expression of the other two NADPH-generating enzymes, malic enzyme 1 (ME1) and isocitrate dehydrogenase 1 (IDH1). The gene expression of phosphoribosyl pyrophosphate amidotransferase (PPAT), which catalyzes the entry into the de novo purine biosynthetic pathway, is also positively regulated by Nrf2, as is the expression of methylenetetrahydrofolate dehydrogenase 2 (MTHFD2), a mitochondrial enzyme with a critical role in providing one-carbon units for de novo purine biosynthesis. Pyruvate kinase (PK) is negatively regulated by Nrf2 and is expected to favor the buildup of glycolytic intermediates and, together with G6PD, metabolite channeling through the pentose phosphate pathway and the synthesis of nucleic acids, amino acids, and phospholipids. Nrf2 negatively regulates the gene expression of ATP-citrate lyase (CL), which may increase the availability of citrate for mitochondrial utilization or (through isocitrate) for IDH1. Red and blue indicate positive and negative regulation, respectively. The mitochondrion is shown in gray. Metabolite abbreviations: G-6-P, glucose 6-phosphate; F-6-P, fructose 6-phosphate; F-1,6-BP, fructose 1,6-bisphosphate; GA-3-P, glyceraldehyde 3-phosphate; 3-PG, 3-phosphoglycerate; PEP, phosphoenolpyruvate; 6-P-Gl, 6-phosphogluconolactone; 6-PG, 6-phosphogluconate; R-5-P, ribulose 5-phosphate; PRPP, 5-phosphoribosyl-α-1-pyrophosphate; THF, tetrahydrofolate; IMP, inosine monophosphate; AMP, adenosine monophosphate; GMP, guanosine monophosphate.

The function of Nrf2 as a master regulator of cellular redox homeostasis is widely recognized. The gene expression of both the catalytic and the regulatory subunits of γ-glutamyl cysteine ligase, the enzyme catalyzing the rate-limiting step in the biosynthesis of reduced glutathione (GSH), is directly regulated by Nrf2 [17]. The xCT subunit of system x_c_^-^, which imports cystine into cells, is also a direct transcriptional target of Nrf2 [18]. In the cell, cystine undergoes conversion to cysteine, a precursor for the biosynthesis of GSH. In addition to its role in GSH biosynthesis, Nrf2 provides the means for the maintenance of glutathione in its reduced state by the coordinated transcriptional regulation of glutathione reductase 1 [19], [20], which reduces oxidized glutathione to GSH using reducing equivalents from NADPH. The required NADPH is provided by four principal NADPH-generating enzymes, malic enzyme 1 (ME1), isocitrate dehydrogenase 1 (IDH1), glucose-6-phosphate dehydrogenase (G6PD), and 6-phosphogluconate dehydrogenase (PGD), all of which are transcriptionally regulated in part by Nrf2 (Fig. 2) [21], [22], [23], [24]. Curiously, Nrf2 also regulates the inducible gene expression of the cytosolic, microsomal, and mitochondrial forms of aldehyde dehydrogenase [25], which use NAD(P)^+^ as a cofactor, giving rise to NAD(P)H. Indeed, the levels of NADPH and the NADPH/NADP^+^ ratio are lower in embryonic fibroblasts isolated from Nrf2-knockout (Nrf2-KO) mice compared to cells from their wild-type (WT) counterparts, and the NADPH levels decrease upon Nrf2 knockdown in cancer cell lines with constitutively active Nrf2 [26]. As expected, the levels of GSH are lower in cells in which Nrf2 has been disrupted; conversely, Nrf2 activation by genetic or pharmacological means leads to GSH upregulation [27], [28], [29]. Importantly, Nrf2 also regulates the gene expression of thioredoxin [30], [31], [32], thioredoxin reductase 1 [28], [29], [32], [33], and sulfiredoxin [34], which are essential for the reduction of oxidized protein thiols.

Given the crucial role of Nrf2 as a master regulator of cellular redox homeostasis, it is not surprising that, compared to WT cells, the levels of reactive oxygen species (ROS) are higher in cells in which Nrf2 has been disrupted (Nrf2-KO) [35]. This difference is particularly striking upon challenge with agents causing oxidative stress. Moreover, cells deficient in Nrf2 are much more sensitive to the toxicity of oxidants of various types and cannot be protected by Nrf2 inducers, which, under the same conditions, provide efficient and long-lasting protection to WT cells [29], [36], [37]. In addition to the overall cellular redox homeostasis, Nrf2 is also critical for the maintenance of the mitochondrial redox homeostasis. Thus, compared to WT, the total mitochondrial NADH pool is significantly increased in Keap1-KO and dramatically decreased in Nrf2-KO cells [35].

Using live cell imaging, we recently monitored the rates of ROS production in primary glioneuronal cocultures and brain tissue slices isolated from WT, Nrf2-KO, or Keap1-knockdown (Keap1-KD) mice [38]. As expected, the rate of ROS production was faster in Nrf2-KO cells and tissues compared to their WT counterparts. However, we made the unexpected observation that, compared to WT, Keap1-KD cells also have higher rates of ROS production, although the magnitude of the difference between the WT and the Keap1-KD genotypes was smaller than that between WT and Nrf2-KO. We then analyzed the mRNA levels of NOX2 and NOX4, the catalytic subunits of the two NADPH oxidase (NOX) isoforms that have been implicated in brain pathology, and found that NOX2 is dramatically increased under conditions of Nrf2 deficiency, whereas NOX4 is upregulated when Nrf2 is constitutively activated, although to a smaller extent. Quantitatively, the magnitude of upregulation in cells and tissues from the mutant mice parallels the corresponding increases in ROS production [38]. Interestingly, not only does Nrf2 regulate NADPH oxidase, but the ROS produced by NADPH oxidase can activate Nrf2, as shown in pulmonary epithelial cells and cardiomyocytes [39], [40]. Furthermore, a very recent study has demonstrated that the NADPH oxidase-dependent activation of Nrf2 constitutes an important endogenous mechanism for protection against mitochondrial damage and cell death in the heart during chronic pressure overload [41].

In addition to the catalytic activity of NADPH oxidase, mitochondrial respiration is another major intracellular source of ROS.By use of the mitochondria-specific probe MitoSOX, we have examined the contribution of ROS of mitochondrial origin to the overall ROS production in primary glioneuronal cocultures isolated from WT, Nrf2-KO, or Keap1-KD mice [38]. As expected, Nrf2-KO cells had higher rates of mitochondrial ROS production than WT. In agreement with the findings for the overall ROS production, the rates of mitochondrial ROS production in Keap1-KD were also higher compared to WT cells. Importantly, blocking complex I with rotenone caused a dramatic increase in mitochondrial ROS production in both WT and Keap1-KD cells, but had no effect in Nrf2-KO cells. In contrast to the expected increase in mitochondrial ROS production in WT cells after addition of pyruvate (to enhance the availability of NADH, increase the mitochondrial membrane potential,and normalize respiration), the production of ROS decreased in Nrf2-KO cells. Together, these findings strongly suggest that, in the absence of Nrf2: (i) the activity of complex I is impaired, (ii) the impaired activity of complex I is due to limitation of substrates, and (iii) the impaired activity of complex I is one of the main reasons for the increased mitochondrial ROS production, possibly owing to reverse electron flow from complex II.

## Nrf2 affects mitochondrial membrane potential and respiration

The mitochondrial membrane potential (Δψ_m_) is a universal indicator of mitochondrial health and the metabolic state of the cell. In a healthy cell, Δψ_m_ is maintained by the mitochondrial respiratory chain. Interestingly, a stable isotopic labeling with amino acids in culture-based proteomics study in the estrogen receptor-negative nontumorigenic human breast epithelial MCF10A cell line has shown that the mitochondrial electron transport chain component NDUFA4 is upregulated by pharmacological activation (by sulforaphane) of Nrf2, whereas genetic upregulation of Nrf2 (by Keap1 knockdown) leads to downregulation of the cytochrome *c* oxidase subunits COX2 and COX4I1 [42]. A study of the liver proteome using two-dimensional gel electrophoresis and matrix-assisted laser desorption/ionization mass spectrometry has found that Nrf2 regulates the expression of ATP synthase subunit α [43]. In addition, the mitochondrial protein DJ-1, which plays a role in the maintenance of the activity of complex I [44], has been reported to stabilize Nrf2 [45], [46], although the neuroprotective effects of pharmacological or genetic activation of Nrf2 are independent of DJ-1 [47]. However, the consequences of these observations for mitochondrial function have not been investigated.

**Fig. 3:**
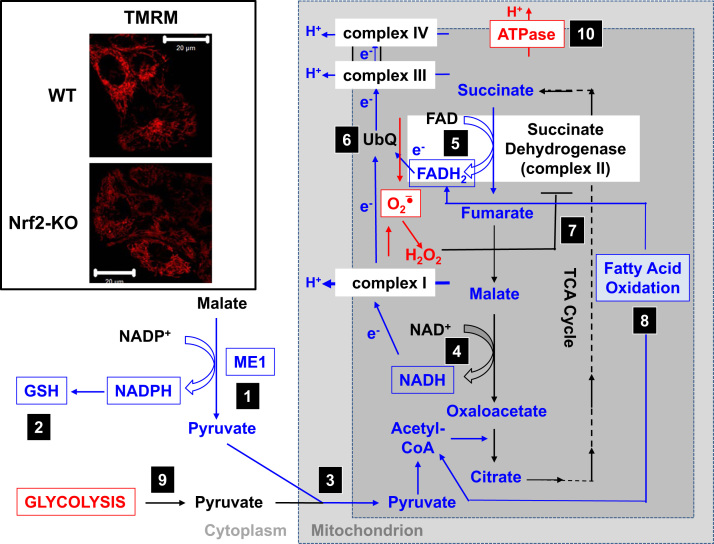
Proposed mechanism for compromised mitochondrial function under conditions of Nrf2 deficiency. (1) The decreased levels of ME1, IDH1, G6PD, and PGD result in lower NADPH levels. (2) The levels of GSH are also low. (3) The low activity of ME1 may decrease the pool of pyruvate entering the mitochondria. (4) The generation of NADH is slower, leading to impaired activity of complex I and increased mitochondrial ROS production. (5) The reduction of FAD to FADH_2_ in mitochondrial proteins is also decreased, lowering the electron flow from FADH_2_ to UbQ and into complex III. (6) The slower formation of UbQH_2_ may lower the enzyme activity of succinate dehydrogenase. (7) The increased levels of ROS may further inhibit the activity of complex II. (8) The lower efficiency of fatty acid oxidation contributes to the decreased substrate availability for mitochondrial respiration. (9) Glycolysis is enhanced as a compensatory mechanism for the decreased ATP production in oxidative phosphorylation. (10) ATP synthase operates in reverse to maintain Δψ_m_. Red and blue indicate upregulation and downregulation, respectively. The boxes signify availability of experimental evidence. The inset shows images of mitochondria of WT and Nrf2-KO cortical astrocytes visualized by the potentiometric fluorescent probe tetramethylrhodamine methyl ester (TMRM; 25 nM). Scale bar, 20 µm.

In agreement with the impaired activity of complex I under conditions of Nrf2 deficiency, the basal Δψ_m_ is lower in Nrf2-KO mouse embryonic fibroblasts (MEFs) and cultured primary glioneuronal cells in comparison with their WT counterparts (Fig. 3,inset) [35]. In contrast, the basal Δψ_m_ is higher when Nrf2 is genetically constitutively upregulated (by knockdown or knockout of Keap1). These differences in Δψ_m_ among the genotypes indicate that respiration is affected by the activity of Nrf2. Indeed, evaluation of the oxygen consumption in the basal state has revealed that, compared to WT, the oxygen consumption is lower in Nrf2-KO and Keap1-KO MEFs, by ~50 and ~35%, respectively.

These differences in Δψ_m_ and respiration among the genotypes are reflected by the rate of utilization of substrates for mitochondrial respiration. Application of substrates for the tricarboxylic acid (TCA) cycle (malate/pyruvate, which in turn increase the production of the complex I substrate NADH) or methyl succinate, a substrate for complex II, causes a stepwise increase in Δψ_m_ in both WT and Keap1-KD neurons, but the rate of increase is higher in Keap1-KD cells. More importantly, the shapes of the response to these TCA cycle substrates are different between the two genotypes, whereby the rapid rise in Δψ_m_ in Keap1-KD cells upon substrate addition is followed by a quick drop rather than a plateau, suggesting an unusually fast substrate consumption. These findings are in close agreement with the much lower (by 50–70%) levels of malate, pyruvate, and succinate that have been observed after a 1-h pulse of [U-^13^C_6_]glucose in Keap1-KO compared to WT MEF cells [24]. In Nrf2-KO neurons, only pyruvate is able to increase the Δψ_m_, whereas malate and methyl succinate cause mild depolarization. The effect of Nrf2 on mitochondrial substrate production seems to be the main mechanism by which Nrf2 affects mitochondrial function. The mitochondrial NADH redox index (the balance between consumption of NADH by complex I and production of NADPH in the TCA cycle) is significantly lower in Nrf2-KO cells in comparison with their WT counterparts, and furthermore, the rates of regeneration of the pools of NADH and FADH_2_ after inhibition of complex IV (by use of NaCN) are slower in the mutant cells.

In mitochondria isolated from murine brain and liver, supplementation of substrates for complex I or for complex II increases the rate of oxygen consumption more strongly when Nrf2 is activated and less efficiently when Nrf2 is disrupted [35]. Thus, malate induces a higher rate of oxygen consumption in Keap1-KD compared to WT, but its effect is weaker in Nrf2-KO mitochondria. Similarly, in the presence of rotenone (when complex I is inhibited), succinate activates oxygen consumption to a greater extent in Keap1-KD compared to WT, whereas the response in Nrf2-KO mitochondria is diminished. In addition, Nrf2-KO primary neuronal cultures and mice are more sensitive to the toxicity of the complex II inhibitors 3-nitropropionic acid and malonate, whereas intrastriatal transplantation of Nrf2-overexpressing astrocytes is protective [48], [49]. Similarly, Nrf2-KO mice are more sensitive to, whereas genetic or pharmacological activation of Nrf2 has protective effects against, neurotoxicity caused by the complex I inhibitor 1-methyl-4-phenylpyridinium ion in the 1-methyl-4-phenyl-1,2,3,6-tetrahydropyridine animal model of Parkinson׳s disease [49], [50], [51], [52], [53], [54], [55], [56], [57], [58], [59], [60], [61].

The respiratory control ratio (RCR), the ratio of State 3 (ADP-stimulated) to State 4 respiration (no ADP present), is decreased in the absence of Nrf2, but the RCR is similar between Keap1-KD and WT mitochondria [35]. As the RCR is an indication of the degree of coupling of the mitochondrial respiratory chain activity to oxidative phosphorylation, this finding indicates that the higher rate of respiration in Keap1-KD mitochondria is not due to uncoupling of oxidative phosphorylation. It further suggests that oxidative phosphorylation is more efficient when Nrf2 is activated. The higher rate of respiration in Keap1-KD mitochondria is consistent with the higher levels of mitochondrial ROS production [38] as higher respiration rates may lead to increased electron leak. However, under conditions of oxidative stress, the increased ROS production is counteracted by the Nrf2-dependent transcriptional upregulation of uncoupling protein 3 (UCP3), which increases the proton conductance of the mitochondrial inner membrane and consequently decreases the production of superoxide [62]. Very recently, it was shown that the lipid peroxidation product 4-hydroxy-2-nonenal mediates the Nrf2-dependent upregulation of UCP3 in cardiomyocytes; this might be particularly important for protection under conditions of oxidative stress such as those during ischemia–reperfusion [63].

## Nrf2 affects the efficiency of oxidative phosphorylation and the synthesis of ATP

In agreement with the effect of Nrf2 on respiration, in brain and liver mitochondria, Nrf2 deficiency results in a decreased efficiency of oxidative phosphorylation (as estimated by the ratio of ADP to oxygen, which is consumed for ATP synthesis), whereas Nrf2 activation (Keap1-KD) has the opposite effect [35]. Compared to WT, the ATP levels are significantly higher in cells with constitutive upregulation of Nrf2 and lower when Nrf2 is knocked down [64] or disrupted [35]. Furthermore, the use of inhibitors of oxidative phosphorylation (oligomycin) or glycolysis (iodoacetic acid) has revealed that Nrf2 changes the way by which cells produce ATP. Thus, in WT neurons, oligomycin causes a complete drop in ATP and iodoacetic acid has no further effect. Remarkably, in Nrf2-KO cells, oligomycin increases the ATP levels, which are then slowly, but completely, depleted by iodoacetic acid, indicating that in the absence of Nrf2, glycolysis, and not oxidative phosphorylation, is the main source of ATP production. Interestingly, despite the increased efficiency of oxidative phosphorylation in Keap1-KD cells, addition of oligomycin results in an ~80% decrease in ATP levels, and iodoacetic acid causes a further ~20% decrease. Thus, either Nrf2 deficiency or its constitutive activation reduces the contribution of oxidative phosphorylation and increases the contribution of glycolysis toward the synthesis of ATP. This effect is particularly pronounced when Nrf2 is absent and is consistent with the dependence of the Δψ_m_ on the presence of glucose in the medium [35] and the increased levels of glycolytic intermediates (G-6-P, F-6-P, dihydroxyacetone phosphate, pyruvate, and lactate) after knockdown of Nrf2 [24].

The increase in ATP levels after inhibition of the F_1_F_0_-ATPase by oligomycin indicates that in the absence of Nrf2, the F_1_F_0_-ATPase functions as an ATPase and not an ATP synthase, i.e., it operates in reverse. Such reversal in activity most likely reflects the need to pump protons across the inner mitochondrial membrane in an attempt to maintain the Δψ_m_, which is crucial for the functional integrity of this organelle. The reversal of the function of the F_1_F_0_-ATPase is also evidenced by the observed mitochondrial depolarization upon oligomycin administration to Nrf2-KO cells, which is in sharp contrast to the hyperpolarization occurring in their WT or Keap1-deficient counterparts [35]. Overall, it seems that under conditions of Nrf2 deficiency ATP is produced primarily in glycolysis, and this ATP is then used in part by the F_1_F_0_-ATPase to maintain the Δψ_m_.

## Nrf2 enhances mitochondrial fatty acid oxidation

The effect of Nrf2 deficiency on the Δψ_m_ is particularly pronounced when cells are incubated in medium without glucose, and the Δψ_m_ is ~50% lower in Nrf2-KO compared to WT cells [35]. Under conditions of glucose deprivation, mitochondrial fatty acid oxidation (FAO) is a major provider of substrates for respiration and oxidative phosphorylation, suggesting that Nrf2 may affect FAO. Indeed, the efficiency of FAO for both the long-chain (C16:0) saturated fatty acid palmitic acid and the short-chain (C6:0) hexanoic acid is higher in Keap1-KO MEFs and isolated heart and liver mitochondria than in their WT counterparts, whereas it is lower in Nrf2-KO cells and mitochondria [65]. These effects are also highly relevant to humans: indeed, metabolic changes indicative of better integration of FAO with the activity of the TCA cycle have been reported to occur in human intervention studies with diets rich in glucoraphanin, the precursor of the classical Nrf2 activator sulforaphane [66].

During the first step of mitochondrial FAO, the pro-*R* hydrogen of the β-carbon leaves as a hydride that reduces the FAD cofactor to FADH_2_, which in turn transfers electrons to ubiquinone (UbQ) in the respiratory chain, ultimately contributing to ATP production. Whereas stimulation of FAO by palmitoylcarnitine in the absence of glucose causes the expected increase in the ATP levels in WT and Keap1-KO cells, with the ATP rise being faster in Keap1-KO cells, the identical treatment produces no ATP changes in Nrf2-KO MEFs [65]. This experiment demonstrates that, in the absence of Nrf2, FAO is suppressed, and furthermore, it implicates suppression of FAO as one of the reasons for the lower ATP levels under conditions of Nrf2 deficiency [35], [64].

Notably, human 293 T cells in which Nrf2 has been silenced have a lower expression of *CPT1* and *CPT2* [67], two isoforms of carnitine palmitoyltransferase (CPT), the rate-limiting enzyme in mitochondrial FAO. In agreement, the mRNA levels of *Cpt1* are lower in livers of Nrf2-KO compared to WT mice [68]. CPT catalyzes the transfer of the acyl group of a long-chain fatty acyl-CoA from coenzyme A to l-carnitine and thus permits the import of acylcarnitine from the cytoplasm into the mitochondria. Although this has not been examined to date, it is possible that in addition to the transcriptional effects on CPT1 expression, Nrf2 may also affect the function of this enzyme by controlling the levels of its main allosteric inhibitor, malonyl-CoA. This is because, by a mechanism that is currently unclear, Nrf2 regulates negatively the expression of stearoyl CoA desaturase (SCD) [69] and citrate lyase (CL) [69], [70]. Curiously, knockout or inhibition of SCD leads to increased phosphorylation and activation of AMP-activated protein kinase (AMPK) [71], [72], [73], and it can be speculated that, in the absence of Nrf2, the SCD levels will increase, in turn lowering AMPK activity. This could be further compounded by the reduced protein levels of AMPK that have been observed in livers of Nrf2-KO mice [68], a finding that is in close agreement with the increased AMPK levels, which have been reported in livers of Keap1-KD mice [74]. One consequence of the decreased AMPK activity is the relief of its inhibitory phosphorylation (at Ser79) of acetyl-CoA carboxylase (ACC) [75], which could be further transcriptionally upregulated in the absence of Nrf2 because it is downregulated by Nrf2 activation [70]. The high ACC activity, in combination with the upregulated CL expression that will increase the production of acetyl-CoA, the substrate for ACC, may ultimately increase the levels of the ACC product, malonyl-CoA. The high levels of malonyl-CoA will inhibit CPT, thereby decreasing the transport of fatty acids into the mitochondria. Finally, Nrf2 positively regulates the expression of CD36 [76], a translocase that imports fatty acids across plasma and mitochondrial membranes. Thus, one mechanism by which Nrf2 may affect the efficiency of mitochondrial FAO is by regulating the import of long-chain fatty acids into the mitochondria.

In addition to direct transcriptional regulation, Nrf2 may also alter the efficiency of mitochondrial FAO by its effects on the cellular redox metabolism. This may be especially relevant when Nrf2 activity is low or absent, conditions that shift the cellular redox status toward the oxidized state. Indeed, several FAO enzymes have been identified as being sensitive to redox changes. One such enzyme is very long-chain acyl-CoA dehydrogenase (VLCAD), which contributes more than 80% to the palmitoyl-CoA dehydrogenation activity in human tissues [77]. Interestingly, Hurd et al. [78] have shown that VLCAD contains cysteine residues that significantly change their redox state upon exposure of isolated rat heart mitochondria to H_2_O_2_. Additionally, S-nitrosylation of murine hepatic VLCAD at Cys238 improves the catalytic efficiency of the enzyme [79], and it is likely that oxidation of the same cysteine may have the opposite effect, ultimately lowering the efficiency of mitochondrial FAO. It is therefore possible that, although the expression levels of VLCAD are not significantly different in WT, Nrf2-KO, or Keap1-KO MEFs [65], the enzyme activity of VLCAD could be lower in the absence of Nrf2 owing to the higher levels of ROS.

Based on all of these findings, it can be proposed that (Fig. 3): in the absence of Nrf2, the NADPH levels are lower owing to decreased expression of ME1, IDH1, G6PD, and PGD. The levels of reduced glutathione are also lower owing to decreased expression of enzymes that participate in its biosynthesis and regeneration and the lower levels of NADPH that are required for the conversion of the oxidized to the reduced form of glutathione. The low expression of ME1 will decrease the pool of pyruvate entering the mitochondria, with glycolysis becoming the major source of pyruvate. The generation of NADH is slower, leading to impaired activity of complex I and increased mitochondrial ROS production. The reduction of FAD to FADH_2_ is also slower, at least in part owing to less efficient fatty acid oxidation, compromising the electron flow from FADH_2_ to UbQ and into complex III. As UbQH_2_ is an activator of succinate dehydrogenase [80], slowing down its formation may lower the enzyme activity of succinate dehydrogenase. The increased levels of superoxide and hydrogen peroxide can inhibit complex II activity further [81]. The lower efficiency of fatty acid oxidation contributes to the decreased substrate availability for mitochondrial respiration and ATP production in oxidative phosphorylation. As a compensatory mechanism, glycolysis is enhanced. ATP synthase functions in reverse, as an ATPase, in an attempt to maintain the Δψ_m_.

## Nrf2 and mitochondrial biogenesis

It has been reported that, compared to WT, the livers of Nrf2-KO mice have a lower mitochondrial content (as determined by the ratio of mitochondrial to nuclear DNA); this is further decreased by a 24-h fast in both WT and Nrf2-KO mice; in contrast, although no different from WT under normal feeding conditions, the mitochondrial content in mice with high Nrf2 activity is not affected by fasting [82]. Interestingly, supplementation with the Nrf2 activator (*R*)-α-lipoic acid [83], [84], [85] promotes mitochondrial biogenesis in 3T3-L1 adipocytes [86]. Two classes of nuclear transcriptional regulators play critical roles in mitochondrial biogenesis. The first class are transcription factors, such as nuclear respiratory factors^1^1 and 2, which control the expression of genes encoding subunits of the five respiratory complexes, mitochondrial translational components, and heme biosynthetic enzymes that are localized to the mitochondrial matrix [88]. Piantadosi et al. [89] have shown that the Nrf2-dependent transcriptional upregulation of nuclear respiratory factor 1 promotes mitochondrial biogenesis and protects against the cytotoxicity of the cardiotoxic anthracycline chemotherapeutic agent doxorubicin. In contrast, Zhang et al. [82] have reported that genetic activation of Nrf2 does not affect the basal mRNA expression of nuclear respiratory factor 1 in the murine liver.

The second class of nuclear transcriptional regulators with critical functions in mitochondrial biogenesis are transcriptional coactivators, such as peroxisome proliferator-activated receptor γ coactivators (PGC)1α and 1β, which interact with transcription factors, the basal transcriptional and RNA-splicing machinery, and histone-modifying enzymes [88], [90], [91]. The expression of the PGC1 family of coactivators is influenced by numerous environmental signals. Treatment of human fibroblasts with the Nrf2 activator sulforaphane causes an increase in mitochondrial mass and induction of PGC1α and PGC1β [92], although the potential dependence on Nrf2 was not examined in this study. However, diabetic mice in which Nrf2 is either activated by *Keap1* gene hypomorphic knockdown (*db/db:Keap1*^flox/−^*:Nrf2*^+/+^) or disrupted (*db/db:Keap1*^flox/−^*:Nrf2*^−/−^) have lower hepatic PGC1α expression levels than control animals (*db/db:Keap1*^flox/+^*:Nrf2*^+/+^) [93]. No differences in the mRNA levels for PGC1α are seen in livers of nondiabetic mice that are either WT or Nrf2-KO, whereas these levels are lower in Nrf2-overexpressing (Keap1-KD and liver-specific Keap1-KO) animals [82]. Notably, a 24-h fast increases the levels of PGC1α mRNA in the livers of mice of all genotypes, but the increase is significantly greater in livers of Nrf2-KO compared to WT or Nrf2-overexpressing mice. Compared to WT, Nrf2-KO mice experiencing septic infection or acute lung injury due to infection show attenuated transcriptional upregulation of nuclear respiratory factor 1 and PGC1α [94], [95]. Together, these observations suggest that the role of Nrf2 in maintaining the levels of both nuclear respiratory factor 1 and PGC1α is complex and becomes most prominent under conditions of stress.

In addition to expression of genes encoding mitochondrial proteins, mitochondrial biogenesis requires the synthesis of nucleotides. Genetic activation of Nrf2 enhances purine biosynthesis by upregulating the pentose phosphate pathway and the metabolism of folate and glutamine, particularly in rapidly proliferating cells (Fig. 2) [24]. Analysis of the transcriptome of mutant *Drosophila* deficient for the mitochondrial serine/threonine protein kinase PTEN-induced putative kinase 1 (PINK1) has shown that mitochondrial dysfunction leads to the transcriptional upregulation of genes affecting nucleotide metabolism [96], suggesting that the enhanced nucleotide biosynthesis represents a mechanism for protection against the neurotoxic consequences of PINK1 deficiency. Nrf2 regulates the expression of phosphoribosyl pyrophosphate amidotransferase (PPAT), which catalyzes the entry into the de novo purine nucleotide biosynthetic pathway, and mitochondrial methylenetetrahydrofolate dehydrogenase 2 (MTHFD2) (Fig. 2). The latter is a bifunctional enzyme with dehydrogenase and cyclohydrolase activities that is critical in providing both glycine and formate as sources of one-carbon units for purine biosynthesis in rapidly growing cells [97]. It is therefore likely that Nrf2 activation might be protective and might reverse mitochondrial dysfunction in PINK1 deficiency. Indeed, pharmacological activation of Nrf2 by sulforaphane, or the triterpenoid RTA-408, restores ∆ψ_m_ and protects PINK1-deficient cells against dopamine toxicity [98]. Although the underlying mechanisms seem to be complex, together, these findings indicate that Nrf2 activity may affect mitochondrial biogenesis by influencing the expression levels of critical transcription factors and coactivators, as well as by enhancing nucleotide biosynthesis.

## Nrf2 and mitochondrial integrity

Although direct evidence is not always available, there are strong indications that Nrf2 is important for mitochondrial integrity, particularly under conditions of oxidative stress. Mitochondria isolated from the brain and liver of rats that had been administered a single dose of the Nrf2 activator sulforaphane are resistant to opening of the mitochondrial permeability transition pore (mPTP) caused by the oxidant *tert*-butylhydroperoxide [99], [100]. The mPTP, a complex that allows the mitochondrial inner membrane to become permeable to molecules with masses up to 1500 Da, was recently identified to be formed from dimers of the F_0_F_1_-ATP synthase [101]. The sulforaphane-mediated resistance to mPTP opening correlates with increased antioxidant defenses, and the levels of mitochondrial GSH, glutathione peroxidase 1, malic enzyme 3, and thioredoxin 2 are all upregulated in mitochondrial fractions isolated from sulforaphane-treated animals [100].

Mitochondrial protein damage and impairment in respiration caused by the electrophilic lipid peroxidation product 4-hydroxy-2-nonenal are attenuated in mitochondria isolated from the cerebral cortex of sulforaphane-treated mice [102]. In rat renal epithelial cells and in kidney, sulforaphane is protective against cisplatin- and gentamicin-induced toxicity and loss of ∆ψ_m_ [103], [104]. Protection against a panel of oxidants (superoxide, hydrogen peroxide, peroxynitrite) and electrophiles (4-hydroxy-2-nonenal and acrolein) and an increase in mitochondrial antioxidant defenses have been also observed upon treatment of rat aortic smooth muscle cells with sulforaphane [105]. In a model of contrast-induced acute kidney injury, limb ischemic preconditioning was recently shown to have protective effects, including inhibition of the opening of the mPTP and mitochondrial swelling, by activation of Nrf2 consequent to the inhibition of GSK3β [106].

Mitophagy, the process by which dysfunctional mitochondria are selectively engulfed by autophagosomes and delivered to lysosomes to be degraded and recycled by the cell, is essential for mitochondrial homeostasis [107], [108]. Whereas no causative relation between Nrf2 and mitophagy has been established, there is evidence that the transcription factor may be important in mitochondrial quality control by playing a role in mitophagy. This might be especially prominent under conditions of oxidative stress. Thus, in a model of sepsis, the increases in the levels of the autophagosome marker MAP1 light chain 3-II (LC3-II) and the cargo protein p62 at 24 h postinfection are suppressed in Nrf2-KO compared to WT mice [109]. A small-molecule inducer of mitophagy (called p62-mediated mitophagy inducer, PMI) was recently discovered; this 1,4-diphenyl-1,2,3-triazole compound was originally designed as an Nrf2 activator that disrupts the interaction of the transcription factor with Keap1 [110]. Similar to cells in which Nrf2 is genetically upregulated (Keap1-KD or Keap1-KO), cells exposed to PMI have higher resting Δψ_m_. Importantly, the increase in mitochondrial LC3 localization that is observed after PMI treatment of WT cells does not occur in Nrf2-KO cells, suggesting the involvement of Nrf2.

Last, ultrastructural analysis of liver sections has revealed the presence of swollen mitochondria with reduced crista and disrupted membranes in hepatocytes of Nrf2-KO, but not WT, mice that had been fed a high-fat diet for 24 weeks; notably, these livers show clear evidence of oxidative stress and inflammation [68]. It can be concluded that Nrf2 has a critical role in maintaining mitochondrial integrity under conditions of oxidative and inflammatory stress.

## Concluding remarks

Although many questions still remain open, the available experimental evidence clearly indicates that Nrf2 is an important player in the maintenance of mitochondrial homeostasis and structural integrity. This role becomes particularly critical under conditions of oxidative, electrophilic, and inflammatory stress when the ability to upregulate Nrf2-mediated cytoprotective responses influences the overall health and survival of the cell and the organism. The role of Nrf2 in mitochondrial function represents another layer of the broad cytoprotective mechanisms orchestrated by this transcription factor. As many human pathological conditions have oxidative stress, inflammation, and mitochondrial dysfunction as essential components of their pathogenesis, pharmacological activation of Nrf2 holds promise for disease prevention and treatment. Comprehensive understanding of the precise mechanisms by which Nrf2 affects mitochondrial function is essential for rational design of future clinical trials and may offer new biomarkers for monitoring therapeutic efficacy.

## Footnotes

1 Unfortunately, the same abbreviation (NRF) is often used to indicate either “nuclear respiratory factor” or “NF-E2 p45-related factor,” as pointed out by Baldelli et al. [87]. Furthermore, there are examples in the literature in which this abbreviation has been used erroneously. As the fields of Keap1–Nrf2 signaling and mitochondrial biology converge, it is of utmost importance to maintain clarity.
